# Post-translational modifications and immune responses in liver cancer

**DOI:** 10.3389/fimmu.2023.1230465

**Published:** 2023-07-11

**Authors:** You-Wei Wang, Jia-Chen Zuo, Chong Chen, Xiao-Hong Li

**Affiliations:** Academy of Medical Engineering and Translational Medicine, Medical College of Tianjin University, Tianjin, China

**Keywords:** hepatocellular carcinoma, post-translational modifications, immune surveillance, phosphorylation, ubiquitination, SUMOylation, glycosylation

## Abstract

Post-translational modification (PTM) refers to the covalent attachment of functional groups to protein substrates, resulting in structural and functional changes. PTMs not only regulate the development and progression of liver cancer, but also play a crucial role in the immune response against cancer. Cancer immunity encompasses the combined efforts of innate and adaptive immune surveillance against tumor antigens, tumor cells, and tumorigenic microenvironments. Increasing evidence suggests that immunotherapies, which harness the immune system’s potential to combat cancer, can effectively improve cancer patient prognosis and prolong the survival. This review presents a comprehensive summary of the current understanding of key PTMs such as phosphorylation, ubiquitination, SUMOylation, and glycosylation in the context of immune cancer surveillance against liver cancer. Additionally, it highlights potential targets associated with these modifications to enhance the response to immunotherapies in the treatment of liver cancer.

## Introduction

Liver cancer is one of the most common malignancies worldwide and directly causes nearly one million deaths each year (1). According to global cancer statistics in 2020, liver cancer is the sixth most diagnosed cancer and the third most common cause of cancer death (2). In 2020, about 900,000 people worldwide were diagnosed with liver cancer and about 800,000 died of liver cancer. It is estimated that the number of liver cancer diagnoses could reach 1.3 million by the year of 2040 (3). Primary liver cancer mainly includes four types: hepatoblastoma (HB), hepatocellular carcinoma (HCC), cholangiocarcinoma (CCA), and combined hepatocellular carcinoma and cholangiocarcinoma (cHCC-CCA) (4). HCC is the main type of primary liver cancer, accounting for approximately 75% of the total number of liver cancer cases worldwide. CCA is the second most common primary liver cancer, of which intrahepatic cholangiocarcinoma (ICC) is a highly heterogeneous primary epithelial liver cancer (5). While novel therapeutic approaches have demonstrated notable clinical efficacy or promising prospects in cancer treatment (6), the current primary approach for liver cancer therapy is still surgical intervention.

Protein translational modifications (PTMs) are covalent attachment of functional groups to protein substrates and can alter the activity, stability, protein interaction, and intracellular localization of target proteins (7). These modifications involve addition of chemical groups (methylation, acetylation, phosphorylation, etc.), addition of polypeptide chains (ubiquitination, SUMOylation, etc.), amino acid modification (racemization, citrullination, etc.), and addition of complex molecules (palmitoylation, oxidation, glycosylation, etc.) (8, 9). PTMs, whether direct or indirect, have a significant impact on the immunogenicity of cancer cells, thereby affecting their recognition and susceptibility to immune system. Furthermore, these modifications also play a crucial role in shaping the response of various immune cells, influencing their interactions with liver tumor cells within the microenvironment. PTMs exert a significant influence on the initiation, progression, immune evasion, and immunotherapy of cancers. By investigating PTMs, we can gain valuable insights into the mechanisms governing cancer-immune cell interactions and potentially develop novel strategies to enhance anti-cancer immune responses.

Acetylation and methylation have received extensive attention in previous reviews (10, 11). In this review, our primary focus will be the profound influence of phosphorylation, ubiquitination, glycosylation, and SUMOylation on liver cancers, with a particular emphasis on their immunological significance.

## Phosphorylation

Phosphorylation, a highly conserved type of PTM (12), primarily targets serine, threonine, or tyrosine residues, and involves a reversible reaction mediated by protein kinases and protein phosphatase (13). This essential modification plays a pivotal role in numerous biological processes, including protein interactions, stability, signal transduction, transcriptional regulation, and intracellular localization (14).

T cells play a central role in the immune system and tumor immune response. Some immunotherapies that target T cells, such as CAR (Chimeric Antibody Receptor)-T cell therapy and checkpoint inhibitors (15, 16), have shown promising results in cancer immunotherapy. T-cell development, differentiation, and activation are intricately regulated by phosphorylation events which target various transcription factors. These phosphorylation events play a critical role in dictating T cell fates and functions. The phosphorylation of specific transcription factors, such as signal transducer and activator of transcription 1 (STAT1) in Th1 cells, STAT6 in Th2 cells, and STAT3 in Th17 cells, contributes to their differentiation and functional specialization (17–21). In patients with HCC, Th1 cytokines of serum level are often suppressed, while Th2 cytokines are frequently elevated (22). Interleukin-6 (IL-6), one of the Th2 cytokines, has been observed to exhibit a negative correlation with overall survival rate and can independently serve as a predictive factor for survival. Conversely, increase of Th1 cytokine responses have been linked to favorable immunological effects on the prognosis of HCC (23). An increase in Th1-related cytokines and a decrease in Th2-related cytokines was observed in a study on primary HCC after radiofrequency ablation (RFA) treatment (22). Th17 cells, a specific subset of T-helper cells, play a pivotal role in immune responses through the production of IL-17 (24, 25). IL-17 acts on HCC cells and triggers the activation of AKT (protein kinase B) through phosphorylation. This activation leads to the production of IL-6 by HCC cells (26). In patients with HCC, there is an elevated presence of Th17 cells compared to healthy individuals, and as the severity of HCC malignancy worsens, the levels of Th17 cells further escalate (27).

Macrophages are the main effector cells in chronic inflammation, a known driver of carcinogenesis (28). Serine/threonine-protein kinase 4 (STK4) was considered as a pivotal tumor suppressor gene in HCC. Notably, significant downregulation of STK4 expression observed in macrophages isolated from HCC patients. This decrease in STK4 expression shows a strong inverse correlation with the levels of IL-1 receptor-associated kinase 1 (IRAK1). Through its interaction with IRAK1 and subsequent phosphorylating it, STK4 exerts inhibitory effects on the secretion of proinflammatory cytokines, including IL-6, IL-1β, and tumor necrosis factor-α (TNF-α), particularly following the activation of Toll-like receptor 4/9 (TLR4/9). This implies that the regulatory mechanism mediated by STK4 attenuates the chronic inflammatory response and significantly reduces the probability of HCC development (29).

Macrophages can be categorized into two subpopulation based on their distinct functions: M1 macrophages, which promote inflammatory responses, and M2 macrophages, which support tissue repair and cell proliferation (30). In liver cancers, macrophages tend to exhibit excessive M2-like polarization, thereby suppressing immune responses against cancer cells. Recent findings highlight the importance of protein phosphorylation in the cancer microenvironment for macrophage polarization (31). Sirtuin 1 (SIRT1) has been shown to enhance the infiltration of M1-like macrophages and inhibit HCC metastasis. This effect is mediated by SIRT1’s ability to enhance nuclear factor kappa-B (NF-κB) activation and promote the phosphorylation of p65, IκB, and IκB kinase (IKK) (22). Zinc finger protein 64 (ZFP64), a gene upregulated in HCC patients with unfavorable prognosis in anti-PD1 treatment, undergoes direct phosphorylation at S226 by protein kinase Cα (PKCα), leading to its nuclear translocation and the transcriptional activation of macrophage colony-stimulating factor (CSF1). CSF1 derived from HCC cells further promotes macrophage polarization towards M2 phenotype.

NK (natural killer) cells earned their name due to their remarkable ability to “naturally” eliminate cancer cells without the need for prior sensitization, and without being restricted by the major histocompatibility complex (MHC) (32). Upon entering the tumor microenvironment (TME) or encountering cancer cells, NK cells can eliminate cancer cells through self-destruction mechanisms (perforin/granzyme mediated) or +antibody-dependent cell-mediated cytotoxicity (ADCC) mechanism (33). In contrast to the NK cells found in peripheral blood, the liver harbors two distinct types of NK cells: one shares similarities with circulating NK cells (cNK cells), while the other primarily resides within liver tissue (trNK cells) (34). Despite various pathways easily active NK cell cytotoxicity, the killing capacity of NK cells can also be easily inhibited, especially within the TME of HCC. The PI3K/AKT/mTOR (phosphoinositide 3-kinase, protein kinase B, and mammalian target of rapamycin) signaling pathway plays a crucial role in the development of HCC and the immune response of NK cells against HCC. Aberrant activation of the PI3K/AKT/mTOR pathway confers HCC cells with enhanced metabolic capacity, promoting their proliferation and metastasis (35). The development and cytotoxic capability of NK cells also heavily rely on the activation of the PI3K/AKT/mTOR signaling pathway (36). PI3K consists of a catalytic subunit, p100, and a regulatory adapter subunit, p85. The p85 subunit is responsible for linking p100 to activated receptor tyrosine kinases (RTKs), thereby activating PI3K and initiating the PI3K/AKT/mTOR signaling pathway (37). Tim-3 is one of the checkpoint molecules expressed on the surface of NK cells. Its expression levels are significantly elevated in HCC. Bind with phosphatidylserine induces phosphorylation of Tim-3, which further interferes with PI3K/AKT/mTOR pathway in NK cells. By competitively binding to p85, phosphorylated Tim-3 reduces the opportunity for PI3K p110 to bind with p85 and leads to decreased activity of the downstream AKT/mTOR pathway, thereby suppressing the activity of liver NK cells, including cNK and trNK (38).

## Ubiquitination and SUMOylation

Ubiquitination is a posttranslational modification wherein ubiquitin molecules are covalently attached to target proteins (39). This process relies on the coordinated action of three key adaptor proteins: ubiquitin activating enzyme (E1), ubiquitin conjugating enzyme (E2), and ubiquitin ligase (E3) (40). The canonical ubiquitination pathway involves the attachment of ubiquitin lysine amino acids (Ub) to glycine residues located at the C-terminus of target proteins, while the atypical pathways involve the conjugation of ubiquitin to cysteine, serine, and threonine residues on target proteins (41). Ubiquitination can facilitate various downstream responses, including degradation, alterations in activity, changes in subcellular localization, or modulation of protein-protein interactions (42–44). Modulating ubiquitin levels has been shown to have a profound impact on T cell activation and can effectively enhance antitumor responses, as indicated by reference (45). Here, we will shift our focus towards the impact of ubiquitination on other immune cells.

IL-2, IL-15, and IL-21 are members of the common gamma chain receptor family cytokines. While they share numerous similarities, these cytokines also show distinct functions within NK cells. IL-15 is primarily involved in promoting NK cell maturation, whereas IL-2 enhances NK cell cytotoxicity (32). IL-21 facilitates NK cell proliferation without causing telomere shortening (46). However, the mechanisms underlying the discriminatory capacity of NK cells among these closely related cytokines, despite their shared receptors, have not been fully elucidated. IL-15 serves as a critical regulator in the development and maturation of NK cells (47), and it has demonstrated the ability to restore NK cell dysfunction that is impaired by HCC (48). Ubiquitination and deubiquitination processes also play vital roles during IL-15-mediated NK cell maturation. Similar to IL-2, IL-15 binds to its receptor trigger not only phosphorylation, but also ubiquitination of AKT. Otub1, a deubiquitinases enzyme, is involved in inhibiting the ubiquitination of AKT. This negative regulation exerted by Otub1 serves as a checkpoint mechanism, influencing the function of NK cells (49). IL-2 and IL-15 share two identical chains in their receptors, and their downstream effects in NK cells are highly similar. However, Otub1 has minimal impact on the activation of AKT by IL-2. Investigating the differential ubiquitination patterns of downstream molecules may provide new insights and potential avenues for fully understanding the function and signal transduction mechanism of these common gamma chain cytokines.

Although the application of CAR-T cell therapy in liver cancer is still in its early stages, it holds tremendous promise for future advancements. A major hurdle in the effectiveness of CAR-T cell therapy lies in the rapid ubiquitination and subsequent degradation of CAR upon interaction with tumor antigens. This phenomenon presents a significant challenge in maintaining the sustained efficacy of CAR-T cell therapy. Fortunately, recent studies have shown that by introducing specific mutations that target the amino acid residues involved in CAR ubiquitination, the long-term killing capacity of CAR-T cells can be significantly improved (50). Ubiquitination is also linked to other protein or gene regulatory mechanisms. For instance, in a study focusing on Treg cells in HCC, it was observed that the expression level of long noncoding RNA lnc-EGFR (Epidermal Growth Factor Receptor) was elevated, showing a positive correlation with tumor size and EGFR/forkhead box protein 3 (Foxp3) expression levels. By directly binding to EGFR protein, lnc-EGFR preventing its ubiquitination and subsequently stabilizing EGFR, thereby enhancing Treg function and promoting the progression of HCC (51).

SUMO (or SUMOylation), which stands for Small Ubiquitin-like Modifier, is a protein modification process that commonly targets lysine residues, involving the attachment of small regulatory peptides of approximately 11 KDa. Like ubiquitin, this post-translational modification regulates various biological processes such as cell division, DNA replication/repair, signal transduction, and cell metabolism (52). HCC-derived exosomes play a significant role in remodeling the TME and promoting HCC progression (53). One key factor involved in this process is the pyruvate kinase M2 isoform (PKM2) found within these exosomes (54, 55). HCC-derived exosomal PKM2 not only induces metabolic reprogramming in monocytes but also triggers the phosphorylation of nuclear STAT3. This phosphorylation leads to the up-regulation of differentiation-associated transcription factors, promoting M2-like macrophage differentiation. The SUMOylation of PKM2 is responsible for its plasma membrane targeting and subsequent excretion through interaction with arrestin-domain-containing protein 1 (ARRDC1). Additionally, the cytokines and chemokines secreted by macrophages further reinforce the association between PKM2 and ARRDC1 in HCC. This reinforcement occurs through a CCL1-CCR8 axis-dependent mechanism, ultimately promoting the excretion of PKM2 from HCC cells. Consequently, a feed-forward regulatory loop is formed, contributing to tumorigenesis (55).

## Glycosylation

Glycosylation is a form of co-translational and post-translational modification that involves the attachment of glycans to proteins. It is primarily categorized into two types: N-chain glycosylation, where the glycan is linked to asparagine residues, and O-chain glycosylation, where the glycan is attached to oxygen atoms on the hydroxyl groups of serine or threonine amino acid residues within protein (56). Many tumor-associated antigens related to HCC are highly glycosylated proteins, and their glycosylation profiles undergo significant changes in HCC patients (56). Aberrant glycosylation not only promotes the proliferation and metastasis of HCC but also plays an important role in immune recognition and immune escape.

Abnormally expressed alpha-fetoprotein (AFP) in HCC has an inhibitory effect on tumor immune surveillance. It has long been observed that AFP in HCC undergoes different glycosylation compared with normal AFP (57). Tumor-derived AFP exhibits stronger immunosuppressive effects, characterized by lower dendritic cell maturation and decreased T cell activation (58). Recent studies using single-cell metabolic profiling and single-cell energetic metabolism by profiling translation inhibition techniques have found that HCC-derived AFP binds significantly more polyunsaturated fatty acids than normal AFP. Phagocytosis of HCC-derived AFP reduced fatty acid uptake by dendritic cells, increased glucose uptake and metabolism, decreased expression of co-stimulatory molecules, and increased expression of immune checkpoint molecules such as PD-L1. These mechanisms help the tumor evade T cell mediated immune surveillance (59).

IL-12 is a cytokine of significant importance in promoting T cell differentiation and IFN-γ production. IL-12 not only activates CD8^+^ T cells and NK cells in HCC tumors (60) but also enhances the cytotoxicity of Glypican-3-targeting CAR-T cells (61). IL-12 (p70) is composed of two subunits, p30 and p40. The free p40 subunit can act as a negative regulator by blocking the binding of IL-12 to its receptor, thereby inhibiting the biological activity of IL-12 (62). The IL-12 cytokine and its family members are glycoproteins (63). Post-translational glycosylation is a critical step in regulating IL-12 secretion (64). Through molecular biology techniques, mutations in the N-glycosylation site (N220) of the p40 subunit, a component of the Th1 cytokine IL-12, have been shown to reduce the secretion of free p40. However, these mutations have minimal impact on IL-12 secretion. As a result, they significantly enhance long-term CD8^+^ T cell responses and provide protection against tumor attacks. These mutations can be utilized as adjuvants to generate long-term memory T cells (65).

Keratinocyte-associated protein 2 (KRTCAP2) is a critical protein involved in N-glycosylation processes, which play a fundamental role in the modification of proteins with complex sugar molecules in various cellular contexts. In HCC, there is a notable upregulation of KRTCAP2 expression, highlighting its potential significance in HCC pathogenesis and progression. Interestingly, high levels of KRTCAP2 are associated with a decreased infiltration of CD8^+^ T cells and CD68^+^ macrophages, both in the tumor region and the surrounding stroma. Furthermore, the expression level of KRTCAP2 shows a negative correlation with the expression of PD-L1 in HCC (66). The interaction between PD-1 and PD-L1 serves as a critical immune checkpoint and has gained significant recognition as a prominent target for cancer immunotherapy. Elucidating the precise role of KRTCAP2 in the modulation of the TME holds considerable scientific significance and translational potential for overcoming immunosuppression in HCC.

**Figure 1:**
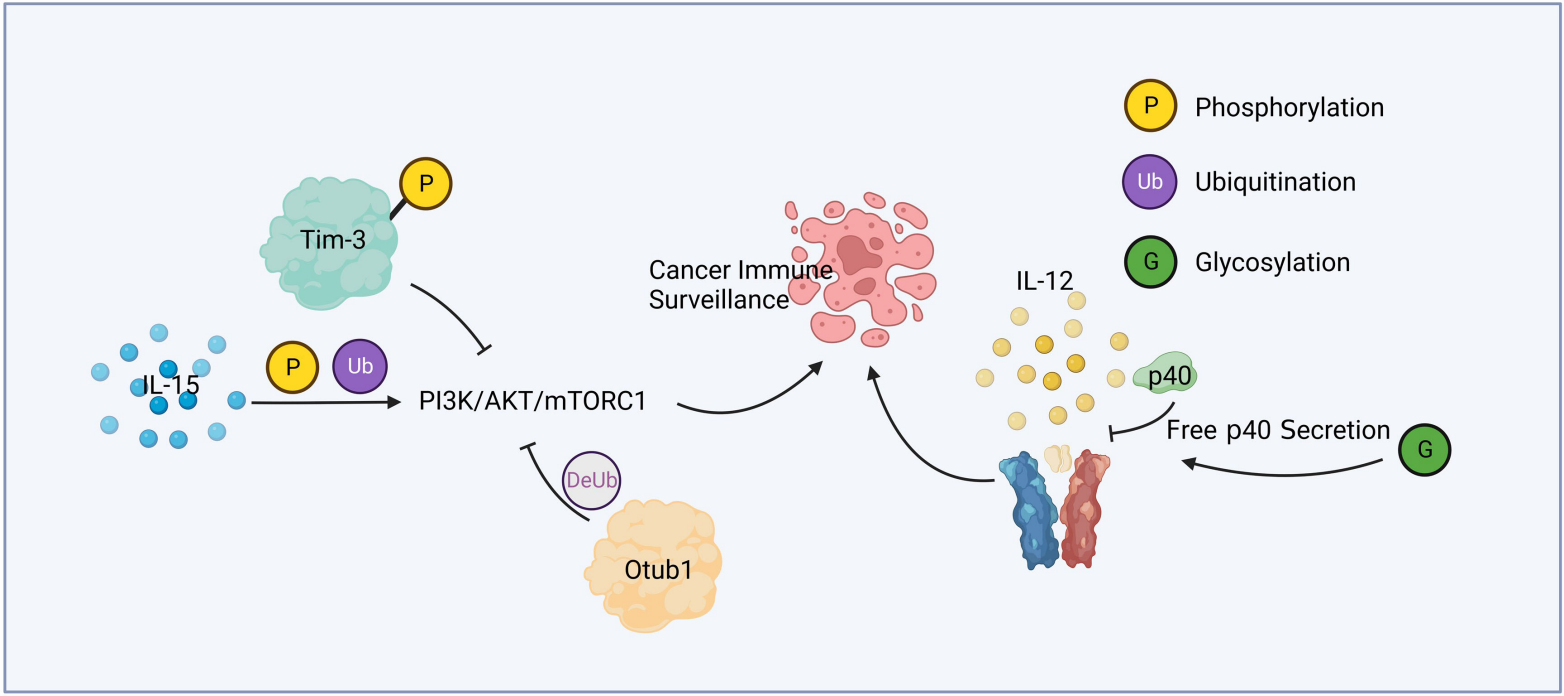
Cytokine relevant post-translational modification and immune surveillance. IL-15 active PI3K/AKT/mTORC1 pathway through phosphorylation and ubiquitination. Phosphorylated Tim-3 competitively inhibits this pathway, while Otub1 downregulates it by deubiquitination. Glycosylation of p40 increases the secretion of free p40, leading to the attenuation of IL-12 signaling.

## Summary and discussion

Liver cancer is a common malignant tumor, which poses a great threat to human health and life. Protein posttranslational modification and immune response play an important role in the development of liver cancer, the immune surveillance against liver cancer, and the treatment of patients with liver cancer. Figure 1 summarized a mechanism by which PTM contributed in cytokine mediated cancer immune surveillance. Numerous studies have shown promising therapeutic potential in targeting PTM for liver cancer treatment. STT3A is a endoplasmic reticulum-associated N-glycosyltransferase, which glycosylates PD-L1 and maintain its stability (67). One notable finding is that spermine, a natural polyamine compound, can activate β-catenin, a protein involved in cell adhesion and signaling pathways. Activation of β-catenin leads to the transcriptional expression of PD-L1 and N-glycosyltransferase STT3A (68). Targeting STT3A might be a potential strategy for improving the response to checkpoint inhibitors in HCC patients.

In the treatment of HCC, certain drugs have been observed to induce alterations in glycosylation. Sorafenib, for instance, has been identified as capable of modifying the glycosylation patterns of multiple proteins in HCC. Further research is needed to determine whether these changes can be targeted to enhance the efficacy of this HCC therapeutic drugs (69). Additionally, researchers are exploring novel approaches that focus on the aberrant glycosylation sites of tumor-associated antigens in HCC. These strategies involve the utilization of antibodies or antigen specific T cells with the aim of converting specific tumor-associated antigens into tumor-specific antigens. Although these studies are still in their early stages, promising preclinical prospects have already emerged (70). Some studies aiming to establish PTM based immunotherapy strategies against HCC were listed in **Table 1**.

**Table 1:** Examples of PTM targeting immunotherapy studies for HCC.

Drug	Immune cells	PTM	Treatment rationale	References
TLR3 agonist with sorafenib	DCs	Phosphorylation	Decreasing phosphorylation of AKT, MEK1/2, ERK1/2 and played an anti-HCC role.	(71)
MY1340	DCs	Phosphorylation	Inhibiting tumor growth *in vivo* by blocking the VEGF-NRP-1 axis through phosphorylation of p65 NF-κB and ERK1/2.	(72)
Caffeic acid (C9H8O4)	Macrophages, T cells	Ubiquitination	Inducing ubiquitination-mediated mortalin degradation to inhibit angiogenesis and reverse sorafenib resistance.	(73)
DMC	CD8^+^T cells	Ubiquitination	Promoting the ubiquitin degradation of PD-L1 in HBx-induced HCC and showing an anti-hepatoma function.	(74)
Targeting MUC1 Glycosylation	CAR-T cells	Glycosylation	Targeting MUC1 aberrant O-glycosylation can control HCC growth.	(75)

MAPK, mitogen-activated protein kinase; ERK, extracellular signal-regulated kinase; MEK, MAPK/ERK kinase; HCC, hepatocellular carcinoma; VEGF, vascular endothelial growth factor; NRP, neuropilin; NF-κB, nuclear factor kappa-B; DMC, 2,5-dimethylcelecoxib; HBx, hepatitis B virus X; MUC1, Mucin1.

In this review, we summarized the current knowledge of post-translational modification of protein in liver cancer cells, tumor infiltrated immune cells, and the microenvironment of liver cancer. Unraveling the intricate network of post-translational modifications in liver cancer holds great promise for advancing our understanding of this disease and undoubtedly contributes to the development of more effective and personalized treatments.

## Author contributions

Y-WW and J-CZ contributed equally to this study. All authors contributed to the article and approved the submitted version.
